# USV Trajectory Tracking Control Based on Receding Horizon Reinforcement Learning

**DOI:** 10.3390/s24092771

**Published:** 2024-04-26

**Authors:** Yinghan Wen, Yuepeng Chen, Xuan Guo

**Affiliations:** 1 School of Automation, Wuhan University of Technology, Wuhan 430070, China; 261688@whut.edu.cn (Y.W.); chenyuepengneu@163.com (Y.C.); 2 School of Information Engineering, Wuhan University of Technology, Wuhan 430070, China

**Keywords:** unmanned surface vehicle, receding horizon reinforcement learning, trajectory tracking, executive–evaluator

## Abstract

We present a novel approach for achieving high-precision trajectory tracking control in an unmanned surface vehicle (USV) through utilization of receding horizon reinforcement learning (RHRL). The control architecture for the USV involves a composite of feedforward and feedback components. The feedforward control component is derived directly from the curvature of the reference path and the dynamic model. Feedback control is acquired through application of the RHRL algorithm, effectively addressing the problem of achieving optimal tracking control. The methodology introduced in this paper synergizes with the rolling time domain optimization mechanism, converting the perpetual time domain optimal control predicament into a succession of finite time domain control problems amenable to resolution. In contrast to Lyapunov model predictive control (LMPC) and sliding mode control (SMC), our proposed method employs the RHRL controller, which yields an explicit state feedback control law. This characteristic endows the controller with the dual capabilities of direct offline and online learning deployment. Within each prediction time domain, we employ a time-independent executive–evaluator network structure to glean insights into the optimal value function and control strategy. Furthermore, we substantiate the convergence of the RHRL algorithm in each prediction time domain through rigorous theoretical proof, with concurrent analysis to verify the stability of the closed-loop system. To conclude, USV trajectory control tests are carried out within a simulated environment.

## 1. Introduction

A USV inherently constitutes a complex nonlinear system, being subject to disturbances and influences from the environment during navigation. Consequently, enhancing the path-tracking accuracy of unmanned ship motion control is a pressing concern.

At present, common methods for achieving such control include the PID [1,2], which is the most widely used, feedback control [3,4], fuzzy control [5,6], module predictive control (MPC) [7,8], and reinforcement learning (RL)-based control [9,10] methods. Of the aforementioned approaches, the PID control method stands out for its advantages. Notably, it eliminates the necessity for modeling the unmanned ship, rendering it a robust and easily implementable controller. However, a challenge lies in ensuring the optimality of specific performance indices. While the fuzzy controller exhibits the capability to deduce and generate expert behavior, its application is challenged by the intricacies of crafting fuzzy rules that primarily arise from the complexity inherent in the navigation environment.

The feedback controller, in its typical operation, computes heading and lateral deviations by analyzing the geometric relationship between the USV and the desired path. Based on this, it directly determines the steering wheel angle for precise steering control. The methods used for tracking, which involve deriving the correlation between the selected path anchor point and the USV position, are the single-point tracking method, pre-sight distance method, and the Stanley method. Both the single-point tracking method [11] and pre-viewing distance method [12,13] offer the advantages of simplicity in algorithms and ease of implementation. However, a notable consideration lies in the fact that the selection of pre-viewing distance is contingent upon the experiential judgment of designers. The Stanley method, initially introduced by Stanford University for an unmanned vehicle fleet, is well suited for lower vehicle speeds. It necessitates a continuous curvature in the reference trajectory for optimal implementation.

A plethora of research findings have emerged concerning the application of MPC in vehicle motion control, as documented in the literature [14,15,16,17]. Of the achievements in these cited works, Falcone et al. [15] introduced an MPC motion controller grounded in the continuous linearization model, and their simulation results underscore the efficacy of the continuous linearization MPC design approach in minimizing computational costs. Carvalho et al. [17] studied an algorithm for local path planning using locally linearized MPC, carrying out linearization and convex approximation of nonlinear obstacle avoidance boundaries. Liniger et al. [18] proposed a lateral motion method of model predictive controlling control (MPCC). Using this method, the lateral deviation is calculated by estimating the position of the projection point, which reduces the computational complexity to a certain extent. Ostafew et al. [19] adopted Gaussian process regression to build a nonparametric model of a mobile robot. In the realm of unmanned surface vehicles, the trajectory tracking controller, employing the MPC method, typically necessitates real-time numerical calculations for solving an open-loop control sequence. The performance of this approach can be influenced by the precision of the model in addition to the unavoidable challenge of managing the complexity inherent in online calculations. Collectively, the current control strategies have various limitations characterized by suboptimal tracking accuracy and constrained computational efficiency.

In recent years, approximate dynamic programming (ADP) as well as reinforcement learning (RL) have experienced widespread adoption in the design of robot decision and control algorithms, thanks to their remarkable efficiency in solving optimization problems and adaptive learning capabilities [20,21]. Yang [22] developed a learning method which is based on PID control for the tracking control of vehicles. Aiming at optimizing the tracking deviation of robots, the DHP algorithm was employed for real-time adjustment of PID parameters, enhancing path-tracking accuracy. Gong et al. [23] designed a finite-time dynamic positioning controller for surface vessels. Shen et al. [24] introduced an innovative LMPC framework aiming to enhance trajectory tracking performance. Jiang et al. [25] also proposed sliding mode control to improve the tracking performance of USVs.

Recent advancements include noteworthy works employing deep learning and deep reinforcement learning to design controllers based on image or state information, facilitating trajectory control for USVs [26,27,28]. A key advantage of this approach lies in leveraging deep networks to enhance the feature representation capabilities of both reinforcement learning and supervised learning. Notably, the training process is entirely data driven, eliminating the need for dynamic model information. However, it has the following disadvantages:

(1) Due to the inherent complexity of deep networks, application of this method is limited to offline training control strategies for online deployment. Moreover, its control performance is susceptible to the influence of factors such as the quantity and distribution of training samples.
(2) In the context of deep network learning, the analysis of theoretical characteristics, such as convergence and robustness, remains a crucial and challenging issue for the academic community to address.

Motivated by the challenges outlined above, we propose a RHRL-based control method, aiming at achieving high-precision lateral control for USVs. The initial step involves constructing a dynamic deviation model for a USV. The steering control of such vehicles comprises two parts, which are feedforward and feedback. Feedforward control is derived directly from the curvature and deviation model for the reference path. In parallel, the establishment of feedback control is achieved by addressing the problem of optimal tracking through application of the RHRL algorithm proposed in this paper. Diverging from conventional optimal control methods rooted in reinforcement learning, RHRL employs a rolling horizon optimization mechanism. This transformation converts infinite time domain optimal control problems into a sequence of finite time domain heuristic dynamic programming problems for resolution. In contrast to the MPC method for unwinding the loop control sequence, the strategy learned by this method is an explicit state feedback control law, which is amenable to offline direct deployment and online learning. Furthermore, in Section 3, the convergence and stability of the closed-loop associated with the proposed RHRL algorithm are theoretically analyzed within each prediction time domain. Finally, simulation and comparative experiments for USV trajectory control using the RHRL algorithm are conducted. Through simulation tests, the control performance is found to be comparable to that of LMPC, with notable advantages in terms of computational efficiency, lower sample complexity, and higher learning efficiency. To verify the algorithm’s robustness and anti-interference capabilities, simulation incorporating disturbances are also conducted.

The remaining sections of this manuscript are arranged as follows. In Section 2, a dynamic model of a USV is built. Then, a USV trajectory control algorithm based on RHRL is proposed and shown to be stable. In Section 3, the simulation and comparison experiments are carried out, and disturbances are added. Section 4 contains the conclusions.

## 2. Materials and Methods

### 2.1. Modeling

In contemporary vehicle modeling, the utilization of three degrees of freedom (DOF) and six DOF predominates. However, considering the environment of the USV investigated in this study, which navigates on the sea surface, we opt for three degrees of freedom in the modeling process to avoid unnecessary complexity.

In the process of establishing dynamical equations, a crucial decision lies in selecting the coordinate system for their formulation. Direct application of Newton’s laws of motion necessitates the expansion of equations in an inertial coordinate system. Nevertheless, various considerations compel us to derive the dynamic equations in a satellite coordinate system. One such reason is to establish dynamic equations that are direction independent. Additionally, employing the satellite coordinate system facilitates the direct assignment of forces and control moments. However, this would result in the current frame of reference not being an inertial frame of reference. Hence, to account for the non-inertial reference frame, Coriolis and centripetal forces are artificially introduced. This allows us to derive the remaining dynamics as if they were in an inertial reference frame.

**Figure 1:**
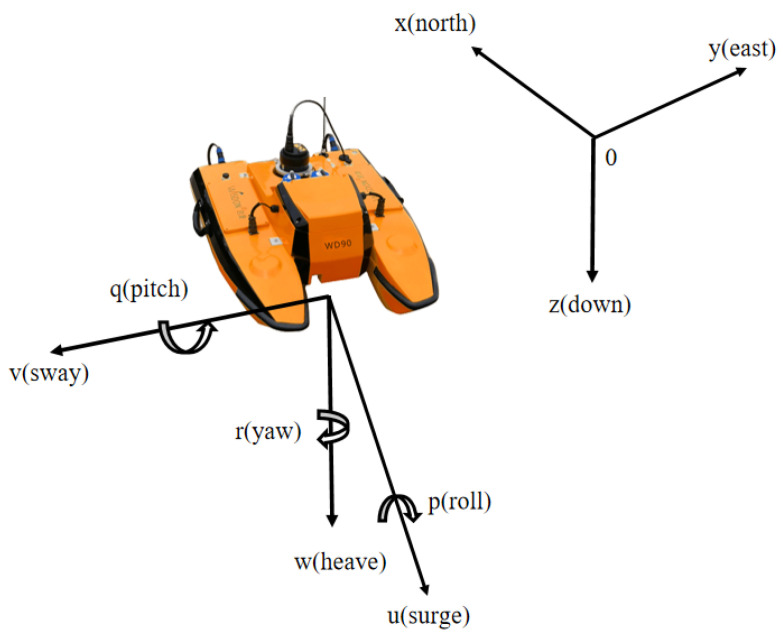
Diagram of the BF (**left**) and IF (**right**).

The USV under investigation features a catamaran-like structure, incorporating two fixed propellers positioned at the extremities of each hull. In Figure 1, variables $U_{1}$ and $U_{2}$ denote the speeds of the two thrusters, while θ represents the heading angle.

Considering its actual working environment, trajectory tracking control of the USV on the horizontal plane will be the focus of our study.

There is a reference frame called the BF (body frame) that is securely fixed to the USV, with the point of origin deliberately chosen to coincide with the center of gravity. Global information is recorded by the IF (inertial frame). Thus, the USV’s motion can be accurately described via the kinematic equation and dynamic equation of the coordinate transformation between these two frames.

The kinematic equation is

(1) $$\dot{\xi }=R\left(\theta \right)v$$

where $\xi ={\left[x,y,z\right]}^{T}$ represents the USV’s position and heading in the IF; $v={\left[u,v,r\right]}^{T}$ represents the USV’s velocity in the BF; and the rotation matrix $R\left(\theta \right)$ depends on θ, which is the heading angle.

$R\left(\theta \right)$ can be expressed by the follow equation:

(2) $$R\left(\theta \right)=\left[\begin{array}{ccc} cos\theta & -sin\theta & 0\\ sin\theta & cos\theta & 0\\ 0 & 0 & 1 \end{array}\right]$$

According to the Newton’s law of motion, the dynamic equation can be established as follows:

(3) $$M\dot{v}+C\left(v\right)v+D\left(v\right)v+g\left(\xi \right)=\kappa$$

where $\kappa ={\left[F_{u},F_{v},F_{r}\right]}^{T}$ represents the thrust force of each propeller. The matrix M takes the mass (which is added) into consideration; $C\left(v\right)$ represents the Coriolis and centripetal matrix. Concrete forms of the above three matrices are shown as follows:

(4a) $$\begin{array}{c} M=\left[\begin{array}{ccc} M_{\dot{u}} & 0 & 0\\ 0 & M_{\dot{v}} & 0\\ 0 & 0 & M_{\dot{r}} \end{array}\right] \end{array}$$

(4b) $$\begin{array}{c} C\left(v\right)=\left[\begin{array}{ccc} 0 & 0 & -M_{\dot{v}}v\\ 0 & 0 & M_{\dot{u}}u\\ M_{\dot{v}}v & -M_{\dot{u}}u & 0 \end{array}\right] \end{array}$$

(4c) $$\begin{array}{c} D\left(v\right)=\left[\begin{array}{ccc} X_{u}+D_{u}\left|u\right| & 0 & 0\\ 0 & Y_{v}+D_{v}\left|v\right| & 0\\ 0 & 0 & Z_{r}+D_{r}\left|r\right| \end{array}\right] \end{array}$$

where $D\left(v\right)$ is the USV’s damping matrix; $g\left(\xi \right)$ denotes the specific restoring force.

The thrusters $\tau ={\left[\tau_{1},\tau_{2},\tau_{3}\right]}^{T}$ generate thrust force κ, and the τ comes from $\kappa =B\left(\alpha \right)\tau$.α denoting the thrusters’ azimuth vector in the BF. We can obtain the distribution of the thruster:

(5) $$\kappa =B\tau ,B=\left[\begin{array}{ccc} 1 & 0 & 1\\ 0 & 1 & 0\\ l_{1} & 0 & l_{2} \end{array}\right]$$

where B denotes an input matrix that is constant. B is a 3×3 matrix that distributes power to the thrusters in three directions, and B satisfies the condition that $B^{T}B$ is not singular. $l_{1},l_{2}\in \left(0,1\right)$ are the thrusters’ efficiency factors.

Therefore, we can derive the dynamic model of the USV for trajectory tracking by combining Equations (1), (3), and (5):

(6) $$\dot{x}=\left[\begin{array}{c} R\left(\theta \right)v\\ M^{-1}B\tau -M^{-1}\mathrm{Cv}-M^{-1}\mathrm{Dv}-M^{-1}g \end{array}\right]=f\left(x,\tau \right)$$

where $x={\left[x,y,\theta ,u,v,r\right]}^{T}$ is the defined state, and input control is expressed as $\tau ={\left[\tau_{1},\tau_{2},\tau_{3}\right]}^{T}$. At the end of this section, we successfully derive the dynamic equation governing USV operation on the water surface.

### 2.2. The USV Trajectory Control Algorithm Based on RHRL

In this section, the USV trajectory control algorithm utilizing RHRL is elaborated. We initially formulate the performance index for the finite time domain trajectory control problem of the USV. Subsequently, we outline the core concepts of the associated reinforcement learning algorithm along with the design and implementation process of the controller. Also included is a detailed analysis of convergence based on this approach.

**Figure 2:**
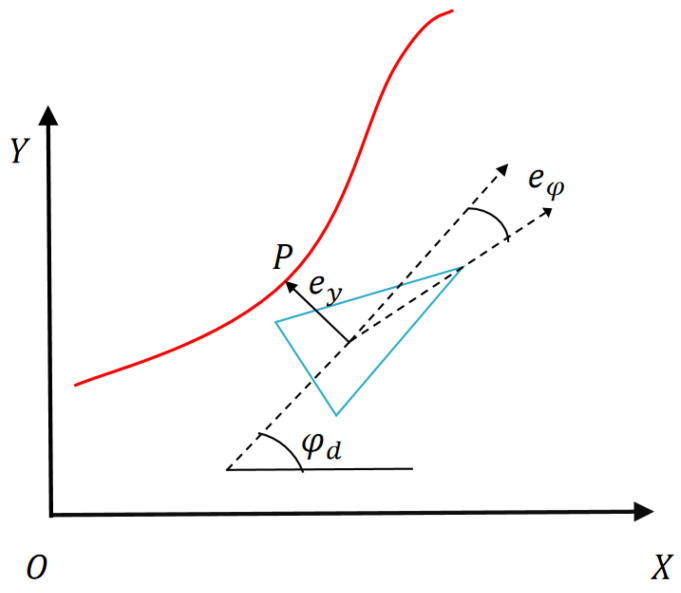
Lateral error model.

When conducting tracking control, it is necessary to describe the relative position between the USV and the desired path, as shown in Figure 2. The point *P* represents the closet point from the desired path, which is called the road projection point. $P(X_{p},Y_{p},\varphi_{d},\kappa )$ is denoted as the path information at the projection point, where $X_{p},Y_{p}$ are the global coordinates of *P*. $\varphi_{d}$ is the angle between the tangent line of *P* and the *X*-axis, also known as the direction of the path; κ is the curvature of the path at point *P*.

The distance between *P* and the USV centroid is called the lateral deviation $e_{y}$, and $e_{y}>0$ is specified for when the USV is located on the left side of the path, and $e_{y}<0$ when the USV is on the right side. Therefore, the lateral deviation can be expressed as

(7) $$e_{y}=-\left(X-X_{p}\right)sin\left(\varphi_{d}\right)+\left(Y-Y_{p}\right)cos\left(\varphi_{d}\right)$$

The path deviation $e_{\varphi }$ of the USV is defined as the difference between the path and the direction, which is $e_{\varphi }=\varphi -\varphi_{d}$. $\varphi =\frac{1}{2}\left(\dot{z}+r\right)tan\theta =rtan\theta$. The first derivative of $e_{y}$ and $e_{\varphi }$ are shown below:

(8) $$\left\{\begin{array}{c} \dot{e_{y}}=v_{y}cos\left(e_{\varphi }\right)+v_{x}sin\left(e_{\varphi }\right)\\ \dot{e_{\varphi }}=\omega -\kappa \left[v_{x}cos\left(e_{\varphi }\right)-v_{y}sin\left(e_{\varphi }\right)\right] \end{array}\right.$$

where $\omega =\dot{\varphi }$. $v_{x}=\dot{x}cos\varphi +\dot{y}sin\varphi +\sqrt{u^{2}+v^{2}}sin\varphi ,v_{y}=-\dot{x}sin\varphi +\dot{y}cos\varphi +\sqrt{u^{2}+v^{2}}cos\varphi$. It is assumed that $v_{x}$ remains constant and there is no sidescale phenomenon in the moving process, and that the expected yaw velocity of the USV’s desired path is constant; then, the lateral acceleration of the USV when it stably tracks the path is $a_{y}={v_{x}}^{2}\kappa$.

Assuming that the course deviation $e_{\varphi }$ is small, then according to the small angle theorem, $sin\left(e_{\varphi }\right)\approx e_{\varphi },cos\left(e_{\varphi }\right)\approx 1$. Then, the second derivative of the lateral deviation with respect to time can be expressed as

(9) $$\ddot{e_{y}}=\left(\dot{v_{y}}+v_{x}\omega \right)-v_{x}^{2}\kappa$$

The first derivative can be approximated as

(10) $$\dot{e_{y}}=v_{y}+v_{x}e_{\varphi }$$

Combining Equations (1), (3) and (4), also (8)–(10); the following equation can be derived as

(11a) $$\begin{array}{c} \dot{e}=A_{c}e+B_{c1}u+B_{c2}\omega_{d} \end{array}$$

(11b) $$\begin{array}{c} A_{c}=\left[\begin{array}{ccc} A_{w} & 0_{6\times 3} & 0_{6\times 3}\\ 0_{3\times 6} & 0_{3\times 3} & I_{3\times 3}\\ 0_{3\times 6} & 0_{3\times 3} & -M^{-1}D \end{array}\right] \end{array}$$

(11c) $$\begin{array}{c} A_{w}=\left[\begin{array}{c} 0_{3\times 3}\\ \bar{A_{w}} \end{array}\right],\bar{A_{w}}=\left[\begin{array}{ccc} M_{\dot{u}}+M_{\dot{v}}V & 0 & X_{u}+D_{u}\left|u\right|\\ 0 & M_{\dot{u}}u & -M_{\dot{r}}\\ M_{\dot{r}} & Y_{v}+D_{v}\left|v\right| & Z_{r}+D_{r}\left|r\right| \end{array}\right] \end{array}$$

(11d) $$\begin{array}{c} B_{c1}=\left[\begin{array}{cc} E_{w} & 0_{6\times 3}\\ 0_{3\times 3} & 0_{3\times 3}\\ 0_{3\times 3} & M^{-1} \end{array}\right],B_{c2}=\left[\begin{array}{c} 0_{6\times 3}\\ 0_{3\times 3}\\ M^{-1} \end{array}\right] \end{array}$$

where $\omega_{d}=\dot{\varphi_{d}},e={\left[e_{y},\dot{e_{y}},e_{\varphi },\dot{e_{\varphi }}\right]}^{T}$, and the control quantity $u=\delta_{f}$.

Given a sampling period Δt, the discrete time model of Equation (11a) can be discretized as

(12) $$e\left(k+1\right)=Ae\left(k\right)+B_{1}u\left(k\right)+B_{2}\omega_{d}\left(k\right)$$

where $A=I+\Delta tA_{c},B_{1}=\Delta tB_{c1},B_{2}=\Delta tB_{c2}$, and *k* is a discrete time point.

**Figure 3:**
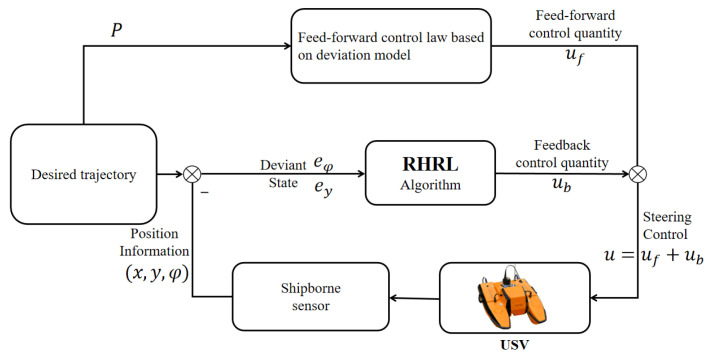
Trajectory tracking control block diagram of the USV.

For the above model Equation (12), it is assumed that path information ${\left(X_{i},Y_{i}\right)}_{i=1}^{M}$, and the purpose of this paper is to design a lateral control algorithm based on RHRL (as shown in Figure 3) such that during the control process, the above-mentioned lateral error state quantity gradually converges to 0, that is, e→0.

#### 2.2.1. Design of Performance Index for the Finite Time Domain Trajectory Control Problem

In this section, a detailed control algorithm based on RHRL is presented. We commence by designing the performance index for the USV finite time domain lateral control problem. Subsequently, we outline the core concept of the RHRL algorithm and delve into the design implementation and convergence analysis based on the actuator–evaluator. For the system deviation model of Equation (12), the control quantity can be decomposed into the form of a feedforward component $u_{f}$ plus a feedback component $u_{b}$ such that $u=u_{f}+u_{b}$, which is shown in Figure 3. The feedforward control quantity represents the expected control input during steady-state vehicle operation and is applicable when the vehicle is stably following the reference path.At the same time e(k)=e(k+1)=0 holds, $u_{b}=0$ as well. The feedforward control quantity $u_{f}$ can be determined as follows:

(13) $$\underset{j=0}{\sum^{\infty }}\;A^{j}B_{1}u_{f}\approx -\underset{j=0}{\sum^{\infty }}\;A^{j}B_{2}\omega_{d}$$

The value in the above formula can be obtained by $\omega_{d}=v_{x}k$. $A,B_{1},B_{2}$ are discrete time coefficient matrices.

Since $u_{f}$ can be easily solved at any current time value *k*, we assume that $u_{f}$ remains constant throughout the prediction time domain [k,k+N], then the feedback control quantity $u_{b}$ to be solved needs to meet the following constraints:

(14) $$u_{b}\in U_{b}=\left\{u\in R\;\;\;|\;\;\;\underline{u}-u_{f}\leq u\leq \bar{u}-u_{f}\right\}$$

where $\bar{u}$ represents the maximum of *u*, $\underline{u}$ is the minimum of *u*. The RHRL algorithm, introduced in this paper, seeks to minimize the following performance indicator function by optimizing $u_{b}\in U_{b}$ in each prediction time domain:

(15) $$V\left(e\left(k\right)\right)=\underset{l=k}{\sum^{k+N-1}}\;L\left(e\left(l\right),u_{b}\left(l\right)\right)+V_{f}\left(e\left(k+N\right)\right)$$

where the cost function $L\left(e\left(l\right),u_{b}\left(l\right)\right)=e^{T}\left(l\right)Qe\left(l\right)+Pu_{b}{\left(l\right)}^{2},Q\in R^{4\times 4}$ is a matrix which is positive definite, *P* is a preset positive real number, and the cost function of the predictive time domain terminal is

(16) $$V_{f}\left(e\left(k+N\right)\right)=e^{T}\left(k+N\right)\bar{R}e\left(k+N\right)$$

where the penalty matrix $\bar{R}\in R^{4\times 4}$ is a positive definite matrix, which can be solved using the following Lyapunov equation:

(17) $$F^{T}\bar{R}F-\bar{R}=-Q-K^{T}PK$$

where $F=A+B_{1}K,K\in R^{1\times 4}$ is the feedback gain matrix satisfying the conditions indicating that *F* is Schur-stable. (The characteristic polynomial ‘F’ for discrete linear systems is such that the roots are located within the unit circle. This property results in the system being classified as Schur-stable).

#### 2.2.2. Path Control Algorithm Based on RHRL

The implementation of the finite time domain reinforcement learning algorithm using the executive–evaluator involves the following main steps:

First of all, according to Equation (15), in any $l\in \left[k,k+N-1\right]$, we can express the value function as a differential form:

(18) $$V\left(e\left(l\right)\right)=L\left(e\left(l\right),u_{b}\left(l\right)\right)+V\left(e\left(l+1\right)\right)$$

where $V\left(e\left(k+N\right)\right)=V_{f}\left(e\left(k+N\right)\right)$. At the *l*-th prediction moment, $V^{*}\left(e\left(l\right)\right)$ would be defined as the optimal value function, and we obtain the HJB equation of the above finite time domain optimization control problem as

(19) $$V^{*}\left(e\left(l\right)\right)=\min_{u_{b}\left(l\right)\in U_{b}}\;L\left(e\left(l\right),u_{b}\left(l\right)\right)+V^{*}\left(e\left(l+1\right)\right)$$

and the optimal control strategy:

(20) $$u^{*}\left(e\left(l\right)\right)=\underset{u_{b}\left(l\right)\in U_{b}}{\mathrm{argmin}}\;L\left(e\left(l\right),u_{b}\left(l\right)\right)+V^{*}\left(e\left(l+1\right)\right)$$

In fact, due to the control constraints, it is difficult to obtain analytical solutions for $V^{*}$ and $u^{*}$ using Equations (19) and (20). In principle, we can approximate the optimal solution of the value function and the control strategy through the method of value iteration. For any $l\in \left[k,k+N-1\right]$, at given initial values where $V^{0}\left(e\left(l\right)\right)=0$, then iterate steps i=0,1,2⋯. This needs to be repeated until $V^{i+1}\left(e\left(l\right)\right)-V^{i}\left(e\left(l\right)\right)\to 0$ to resolve the following two steps.

(1) Strategy update
  (21a) $$\begin{array}{c} u^{i}\left(e\left(l\right)\right)=\underset{u_{b}\left(l\right)\in U_{b}}{\mathrm{argmin}}\;L\left(e\left(l\right),u_{b}\left(l\right)\right)+V^{i}\left(e\left(l+1\right)\right) \end{array}$$
(2) Value update
  (21b) $$\begin{array}{c} V^{i+1}\left(e\left(l\right)\right)=L(e\left(l\right),u_{b}^{i}\left(e\left(l\right)\right)+V^{i}\left(e\left(l+1\right)\right) \end{array}$$

In conclusion, the task of trajectory tracking is accomplished through continuous updating of the strategy and feedback values.

#### 2.2.3. Rolling Time Domain Executor–Evaluator Learning Implementation

We employ the executive–evaluator structure to implement the finite time domain value function iteration algorithm described above. In existing finite time domain reinforcement learning control algorithms [17], the value function in the prediction time domain is regarded as a time-dependent function.

**Assumption 1.**  *If there has a control strategy $u_{b}\left(e\right)=\Phi \left(v\left(e\right)\right)$ so that system (Equation (12)) is asymptotically stable under control strategy $u=u_{b}+u_{f}$, where $\Phi \left(v\left(e\right)\right)$ is a continuous function satisfying $u_{b}\left(e\right)\in U_{b},\forall v\left(e\right)\in R$.*

The aforementioned assumptions essentially represent another aspect of the stabilizability of the system Equation (12). Simultaneously, it is worth noting that the dynamic model Equation (12) presented in this paper is controllable, so there must be a continuous equation $u_{b}\left(e\right)\in U_{b}$ that renders Equation (16) asymptotically stable under the control strategy $u=u_{b}+u_{f}$. Therefore, the above assumptions are reasonable.

We define $\chi_{f}$ as a control invariant set under the control law $u_{b}=Ke\in U_{b}$, then we can state the following theorem.

**Theorem 1.**  *(Time-independent value function) If the value of the prediction time domain N satisfies $t\in \left[k,k+N\right]$ in any prediction time domain, for any initial state $e\left(k\right)\in R^{4}$, the terminal state $e\left(k+N\right)\in \chi_{f}$ is driven by the control strategy $u\left(e\left(l\right)\right),l\in \left[k,k+N-1\right]$ of system Equation (9) such that there is such a control strategy $u_{b}\left(e\right)\in U_{b}$ that $V\left(e\left(l\right)\right)$, and $l\in \left[k,k+N-1\right]$ is a function that is independent of time.*

**Proof of Theorem 1.**  Firstly, consider the case of $e\left(k\right)\in \chi_{f}$. Based on the definition of $\chi_{f}$, there is a control law $u_{b}=Ke=\Phi \left(v\left(e\right)\right)\in U_{b}$ that ensures the quantity of states at any time in the future satisfy $x\left(l\right)\in \chi_{f}$. From that, we can solve and obtain the following function:

(22) $$V\left(e(l)\right)=\sum_{i=l}^{k+n-1}L\left(e\left(i\right),u_{b}\left(i\right)\right)+V_{f}\left(e(k+N)\right)=e{\left(l\right)}^{T}\bar{P}e\left(l\right)$$

For the case of $e\left(k\right)\notin \chi_{f}$, according to Assumption 1, there is such a control strategy $u_{b}=\Phi \left(v\left(e\right)\right)$ and a finite prediction step *N* that $e\left(k+N\right)\in \chi_{f}$. In particular, let v=Ke, then

(23) $$V\left(e\left(l\right)\right)=\underset{i=l}{\sum^{k+n-1}}\;L\left(e\left(i\right),u_{b}\left(i\right)\right)+V_{f}\left(e\left(k+N\right)\right)=\underset{i=l}{\sum^{+\infty }}\;L\left(e\left(i\right),u_{b}\left(i\right)\right)$$

where $u_{b}=\Phi \left(v\left(e\right)\right)$.

Hence, a value function and a strategy independent of time exist. Drawing inspiration from this, we adopt a time-independent executive–evaluator structure to execute the finite time-domain value function iteration process described above. Initially, a network of evaluators is designed to approximate the value function:

(24) $$\hat{V}\left(e\right)={{\hat{W}}_{c}}^{T}\varphi \left(e\right)$$

where $\hat{W_{c}}\in R^{N_{c}}$ represents the weight of the evaluator network, $N_{c}$ denotes network node number; $\varphi \left(e\right)$ is the network’s basis function. According to the definition of the evaluator network, the resulting errors *E* and the end error $E_{f}$ can be expressed as

(25) $$E\left(l\right)={\hat{W_{c}}}^{T}\varphi \left(l\right)-L\left(e\left(l\right),\hat{u_{b}}\left(l\right)\right)-{\hat{W_{c}}}^{T}\varphi \left(l+1\right)$$

(26) $$E_{f}={\hat{W_{c}}}^{T}\varphi \left(e_{f}\right)-{e_{f}}^{T}\bar{P}e_{f}$$

Therein, $e_{f}=e\left(k+N\right)$, which can be randomly valued around 0. By minimizing $E_{c}\left(l\right)=E{\left(l\right)}^{2}+{E_{f}}^{2}$, the equation for updating the weights of the evaluator network is derived as follows:

(27) $$\hat{W_{c}}\left(l+1\right)=\hat{W_{c}}\left(l\right)+\mu_{c}\left(\Delta \varphi \left(e\left(l+1\right)\right)E\left(l\right)-\varphi \left(e_{f}\right)E_{f}\right)$$

where $\mu_{c}>0$ is the learning rate of the evaluator network.

Next, to deal with control constraints, we construct the network of actuators as follows:

(28) $$\hat{u_{b}}\left(l\right)=\bar{u_{1}}tanh\left({\hat{W_{a}}}^{T}\sigma \left(e\left(l\right)\right)\right)+\bar{u_{2}}$$

where $\hat{u_{1}}=0.5\left(\bar{u_{b}}-\underline{u_{b}}\right)$, $\hat{u_{2}}=0.5\left(\bar{u_{b}}+\underline{u_{b}}\right)$, including $\hat{W_{a}}\in R^{N_{a}}$ is the weight of actuator network; $\sigma \left(e\right)$ is the basis function vector of the network. $N_{a}$ indicates the node number, which is on network. Given that the actuator network aims to approximate the optimal strategy of control, we define the control quantity deviation as follows:

(29) $$E_{a}\left(l\right)={\hat{W_{a}}}^{T}\sigma \left(e\left(l\right)\right)+\frac{1}{2}R^{-1}{B_{1}}^{T}\nabla \varphi \left(e\left(l\right)\right)\hat{W_{c}}\left(l\right)$$

By minimizing ${E_{a}}^{2}$, we can obtain the update rule of the network weight as

(30) $$\hat{W_{a}}\left(l+1\right)=\hat{W_{a}}\left(l\right)-\mu_{a}\frac{\delta {E_{a}}^{2}\left(l\right)}{\delta \hat{W_{a}}\left(l\right)}$$

where $\mu_{a}>0$ represents the learning rate of the actuator network.
**Algorithm 1** The main steps of implementing the above finite time domain reinforcement learning algorithm, which makes use of the executive–evaluator.(I)Initialize the weights $\hat{W_{c}}$, $\hat{W_{a}}$, and obtain the initial state $Z\left(0\right)$.(II)When the time t=kΔt, the projection point *P* is found according to the state $Z\left(t\right)$, and the deviation state $e\left(t\right)$ is calculated.(III)$\forall l\in \left[k,k+N-1\right]$, repeat the following process 1–3:(1)According to Equations (17) and (28), $u_{f}\left(l\right)$ and $\hat{u_{b}}\left(l\right)$ are respectively calculated.(2)Update $\hat{W_{c}}$, $\hat{W_{a}}$ according to Equations (27) and (30).(3)Calculate $u\left(l\right)=u_{f}\left(l\right)+\hat{u_{b}}\left(l\right)$ according to Equations (13) and (28), and apply the prediction model for $e\left(l+1\right)$.(IV)Calculate $u_{f}\left(k\right)$ and $\hat{u_{b}}\left(e\left(k\right)\right)$ according to Equations (12) and (27), respectively.(V)In the time period $\left[k\Delta t,\left(k+1\right)\Delta t\right]$, apply quantity $u\left(t\right)=u\left(k\Delta t\right)$ directly to the USV, and update the system states $Z\left(\left(k+1\right)\Delta t\right)$.(VI)Set k←k+1 and repeat operations II-V based on the receding time domain optimization strategy. □

#### 2.2.4. Convergence Analysis of the Weight of Finite Time Domain Actuator and Evaluator

Next, we present the convergence analysis of the above RHRL algorithm in each prediction domain $\left[k,k+N-1\right]$. First, the (local) optimal value function and control strategy can be represented as a network:

(31) $$V^{*}\left(e\right)={W_{c}}^{T}\varphi \left(e\right)+\kappa_{c}$$

(32) $${u_{b}}^{*}=\bar{u_{1}}\tan h\left({W_{a}}^{T}\mu \left(e\right)+\kappa_{a}\right)+\bar{u_{2}}$$

where both $W_{a}$ and $W_{c}$ are weight matrices, and $\kappa_{a}$ and $\kappa_{c}$ are the errors of reconstruction.

**Assumption 2.**  *(Network reconstruction error)*

*(1)* 
$W_{c}\leq W_{c,m},\varphi \leq \varphi_{m},\nabla \varphi \leq {\bar{\varphi }}_{m},\kappa_{c}\leq \kappa_{c,m},\nabla \kappa_{c}\leq {\bar{\kappa }}_{c,m}$

*(2)* 
$W_{a}\leq W_{a,m},\psi \leq \psi_{m},\kappa_{a}\leq \kappa_{a,m}$

**Assumption 3.**  *(Continuous excitation)*

*There are positive real numbers* $q_{1},q_{2},\left(q_{1}<q_{2}\right)$ *such that*

(33) $$q_{1}\leq \bar{\varphi },{\bar{\varphi }}_{f}\leq q_{2}$$

*where $\bar{\varphi }=\Delta \varphi^{T}\Delta \varphi ,{\bar{\varphi }}_{f}={\varphi_{f}}^{T}\varphi_{f},\varphi_{f}=\varphi \left(e_{f}\right)$.*

*In order to more compactly describe the following theorem, define* $\gamma_{1}=4-4\bar{\psi }\mu_{a}-\left(4-8\bar{\psi }\mu_{a}\right)$$\left(\beta_{1}+\beta_{3}\right),\bar{\psi }=\psi^{T}\psi ,\bar{\varphi }=\bar{\varphi }\left(l+1\right)+{\bar{\varphi }}_{f},\alpha =\beta_{0},\beta_{1},\beta_{2},\beta_{3}$ *are tunable positive real numbers.*

**Theorem 2.**  *Under Assumptions 2 and 3, if the appropriate learning laws $\mu_{c}$ and $\mu_{a}$ and ${\left\{\beta_{i}\right\}}^{3}\left(i=0\right)$ are chosen so that $\gamma_{1}>0$ and $\alpha -\gamma_{2}>0$, then the network weights ${\hat{W}}_{c}$ and ${\hat{W}}_{a}$ of Equations (27) and (30) will asymptotically converge to the following region when using the above strategy:*

(34a) $$\begin{array}{c} {\bar{W}}_{c}\leq \frac{\sqrt{E_{t}}}{\sqrt{\gamma_{1}}} \end{array}$$

(34b) $$\begin{array}{c} \varepsilon_{a}\leq \frac{\sqrt{E_{t}}}{\sqrt{\alpha -\gamma_{2}}\lambda_{min\left(\bar{g}\right)}} \end{array}$$

*where ${\bar{W}}_{c}=W_{c}-{\hat{W}}_{c},{\bar{W}}_{a}=W_{a}-{\hat{W}}_{a},\xi_{a}={\bar{W}}_{a}^{T}\psi$, and $E_{t}$ is the error.*

*Furthermore, if $\kappa_{c,m},{\bar{\kappa }}_{c,m},\kappa_{a,m}\to 0$, then ${\bar{W}}_{c}$ and $\xi_{a}$ converge asymptotically to 0.*

**Proof of Theorem 2.**  The Lyapunov function is defined as follows:

$$L\left(l\right)=L_{c}\left(l\right)+L_{a}\left(l\right)$$

where $L_{c}=tr\left({\bar{W}}_{c}^{T}{\eta_{c}}^{-1}{\bar{W}}_{c}\right)$, and $L_{a}=tr\left({\bar{W}}_{a}^{T}{\eta_{a}}^{-1}{\bar{W}}_{a}\right)$. They can be calculated based on Equation (26).

(35) $$E\left(l\right)={\hat{W}}_{c}^{T}\varphi \left(l\right)-{\hat{W}}_{c}^{T}\varphi \left(l+1\right)+\Delta V^{*}\left(l+1\right)={\bar{W}}_{c}^{T}\Delta \varphi \left(l+1\right)+\Delta \kappa_{c}\left(l+1\right)$$

where $\Delta V^{*}\left(l+1\right)=V^{*}\left(l+1\right)-V^{*}\left(l\right),\Delta \kappa_{c}\left(l+1\right)=\kappa_{c}\left(l+1\right)-\kappa_{c}\left(l\right)$.

(36) $$E_{f}={\bar{W}}_{c}^{T}\varphi_{f}-W_{c}^{T}\varphi_{f}-\kappa_{c,f}=-{\bar{W}}_{c}^{T}\varphi_{f}-\kappa_{c,f}$$

where $\kappa_{c_{1}}f=\kappa_{c}\left(k+N\right)$, then according to Equations (27), (35) and (36):

(37) $$\begin{array}{cc} \Delta L_{c}\left(l+1\right) & =L_{c}\left(l+1\right)-L_{c}\left(l\right)\\ \; & =2{\bar{W}}_{c}^{T}\left(-\bar{\varphi }{\bar{W}}_{c}+{\bar{\kappa }}_{c}\right)+\mu_{c}{\left(-\bar{\varphi }{\bar{W}}_{c}+{\bar{\kappa }}_{c}\right)}^{T}\left(-\bar{\varphi }{\bar{W}}_{c}+{\bar{\kappa }}_{c}\right)\\ \; & \leq -\alpha {{\bar{W}}_{c}}^{2}+E_{c} \end{array}$$

where ${\bar{\kappa }}_{c}=-\Delta \varphi \left(l+1\right)\Delta \kappa_{c}\left(l+1\right)-\varphi_{f}\kappa_{cf}$, $E_{c}=\left(2\mu_{c}+{\beta_{0}}^{-1}\right){{\bar{\kappa }}_{c}}^{2}$.

Similarly, $\Delta L_{a}\left(l+1\right)$ can be expressed as

$$\Delta L_{a}\left(l+1\right)=tr\left[2{\bar{W}}_{a}^{T}\left(l\right)\frac{\partial {E_{a}}^{2}\left(l\right)}{\partial {\hat{W}}_{a}\left(l\right)}+\mu_{a}{\left(\frac{\partial {E_{a}}^{2}\left(l\right)}{\partial {\hat{W}}_{a}\left(l\right)}\right)}^{T}\frac{\partial {E_{a}}^{2}\left(l\right)}{\partial {\hat{W}}_{a}\left(l\right)}\right]$$

In consideration of $E_{a}=-\xi_{a}-g{\bar{W}}_{c}+{\bar{\kappa }}_{a}$, $g=\nabla \varphi \left(\frac{1}{2}R^{-1}{B_{1}}^{T}\right)$, ${\bar{\kappa }}_{a}=-\kappa_{a}-\nabla \kappa_{c}\left(\frac{1}{2}R^{-1}{B_{1}}^{T}\right)$, and $\frac{\partial {E_{a}}^{2}\left(l\right)}{\partial {\hat{W}}_{a}\left(l\right)}=2\psi E_{a}$, then

$$\Delta L_{a}=-\left(4-4\bar{\psi }\mu_{a}\right)∥\xi_{a}{∥}^{2}-8\bar{\psi }\mu_{a}g{\bar{W}}_{c}{\bar{\kappa }}_{a}+4\bar{\psi }\mu_{a}∥{\bar{W}}_{c}∥{\bar{g}}^{2}+\left(4-8\bar{\psi }\mu_{a}\right)\left(\xi_{a}{\bar{\kappa }}_{a}-\xi_{a}^{T}g{\bar{W}}_{c}\right)$$

where $\bar{g}=g^{T}g$. According to Young’s inequality theorem,

$$\Delta L_{a}\left(l+1\right)\leq -\gamma_{1}∥\xi_{a}{∥}^{2}+\gamma_{2}{∥{\bar{W}}_{c}∥}_{\bar{g}}^{2}+E_{a}$$

where $E_{a}=\left(1/\beta_{2}+1/\beta_{3}\right){{\bar{\kappa }}_{a}}^{2}$. Then, by defining $E_{t}=E_{c,m}+E_{a,m}$, we obtain

(38) $$\Delta L=-\gamma_{1}∥\xi_{a}{∥}^{2}-\left(\alpha -\gamma_{2}\right){∥{\bar{W}}_{c}∥}_{\bar{g}}^{2}+E_{t}$$

On this basis, if ${\bar{\kappa }}_{c,m}\;,\;\kappa_{c,m}\;,\;\kappa_{a,m}\to 0$, $E_{t}\to 0$ is obtained, then ${\bar{W}}_{c}$ and $\xi_{a}$ asymptotically converge to 0.

Hence, at this juncture, we have successfully concluded the proof of Theorem 2. □

The conclusion of the above theorem indicates that we can make u converge to $u_{b}^{*}$ with an arbitrarily small error by increasing the number of base function nodes in the actuator and the evaluator. Therefore, under the premise that Assumption 1 is true, if a sufficiently large *N* is chosen, the equation of system (12) satisfies the terminal state $e\left(k+N\right)\in x_{f}$ in the prediction time domain $\left[k,k+N-1\right]$ driven by strategy $u_{b}^{*}\left(k\;\;\;|\;\;\;k\right),\cdots$, $u_{b}^{*}\left(k+N-1\;\;\;|\;\;\;k\right)$. Thus, the next prediction time domain $\left[k+1,k+N\right]$, $u_{b}^{*}\left(k+1\;\;\;|\;\;\;k\right),\cdots ,u_{b}^{*}\left(k+N-1\;\;\;|\;\;\;k\right)$, $Ke\left(k+N\;\;\;|\;\;\;k\right)$ is a feasible control strategy. We define the loss function produced by the feasible strategy for $Los^{f}\left(k+1\;\;\;|\;\;\;k\right)$, and referring to Rawling’s [29], $Los^{f}\left(k+1\;\;\;|\;\;\;k\right)-Los^{*}\left(k\;\;\;|\;\;\;k\right)\leq -L\left(e\left(k\;\;\;|\;\;\;k\right),u_{b}\left(k\;\;\;|\;\;\;k\right)\right)$ is available. Due to $Ke\left(k+N\;\;\;|\;\;\;k\right)$ being suboptimal, we may safely derive

$$Los^{*}\left(k+1\;\;\;|\;\;\;k+1\right)-Los^{*}\left(k\;\;\;|\;\;\;k\right)\leq Los^{f}\left(k+1\;\;\;|\;\;\;k\right)-Los^{*}\left(k\;\;\;|\;\;\;k\right)\leq -L\left(e\left(k\;\;\;|\;\;\;k\right),u_{b}\left(k\;\;\;|\;\;\;k\right)\right)$$

which can be obtain by using Lyapunov stability analysis of the stability of the system, which is a closed-loop system.

## 3. Simulation Analysis

To ensure a precise comparison of the control performance between RHRL, Lyapunov-based MPC (LMPC), and sliding mode control (SMC), the control variable method was adopted using experimental parameters from [24,25]. In the simulations, all of the hydrodynamic parameters in the equations are based on the Falcon model [30].

The simulation results are presented in this section in showcasing the advantages of the RHRL method. In addition, the operating environment is Matlab 2021b, and the core is R7-5800H.

### 3.1. Parameter Selection

Two distinct desired trajectories are employed. Refer to the article of Li [31], where one trajectory (Path I) is a typical sinusoidal path:

(39) $$\left.p\left(t\right)=\left\{\begin{array}{c} x_{d}=0.4t\\ y_{d}=\sin\left(0.4t\right) \end{array}\right.\right.$$

The other trajectory, Path II, is based on [32] and is an S-shaped path:

(40) $$\left.p\left(t\right)=\left\{\begin{array}{c} \chi_{d}=-\sin\left(0.4t\right)\\ y_{d}=\sin\left(0.24t\right) \end{array}\right.\right.$$

For the RHRL controller, the following parameters are utilized: the prediction horizon is set such that T=5δ, where δ=0.1 [s] represents the time period; three matrices are set for weighting as $Q=diag(10^{5},10^{5},10^{3},10^{2},10^{2},10^{2})$, $R=diag(10^{-4},10^{-4},10^{-4},10^{-4})$, and $P=diag(10^{3},10^{3},10^{2},10,10)$. The gains of the control are $K_{p}=K_{d}=diag\left(1,1,1\right)$.And the $l_{1}=l_{2}=0.8$.

In this section, the desired trajectory tracking simulation of a USV based on RHRL will be executed as described to emphasize the feasibility and efficiency of RHRL algorithm proposed earlier. The parameters for USV simulation are presented in Table 1.

### 3.2. Tracking Performance

**Figure 4:**
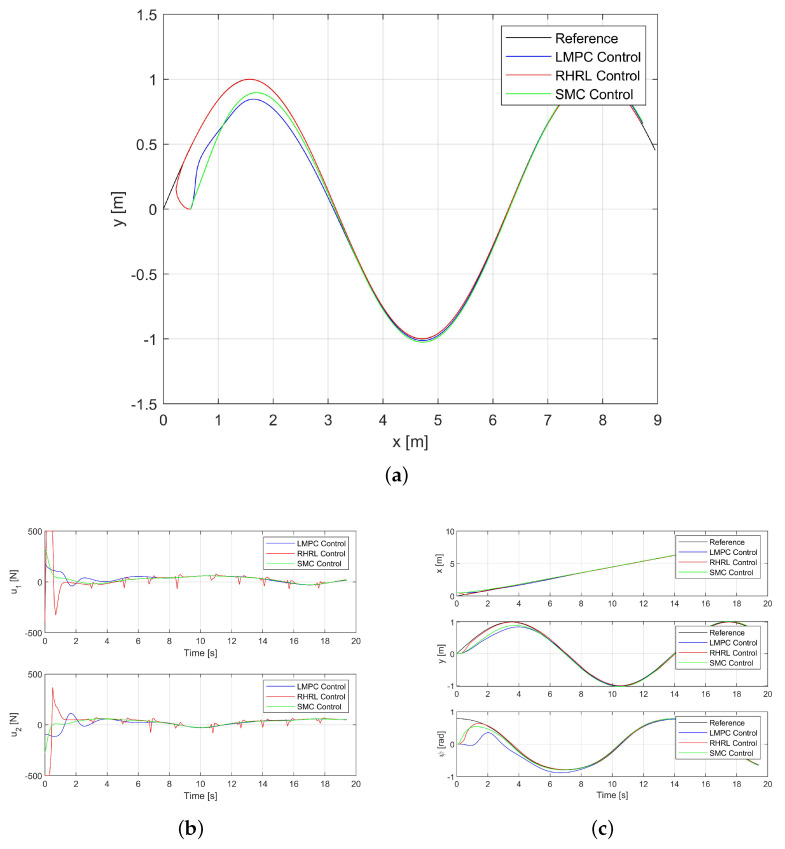
The USV trajectory tracking performance in Path I. (**a**) The USV trajectory for Path I. (**b**) The thrust outputs for Path I. (**c**) The state trajectories for Path I.

Both Figure 4a,c depict the tracking results for Path I. The USV trajectories are represented by the blue curve for the LMPC control method, the green curve for the SMC controller, and the red curve for the USV RHRL controller, and the black curve illustrates the sinusoidal trajectory, which is the desired trajectory. The results demonstrate that all controllers are successful in guiding the USV along the desired trajectory, affirming the stability of the closed loop. However, the RHRL method notably exhibits a considerably accelerated convergence compared to the LMPC and SMC methods. This acceleration in convergence is attributed to the selection of control gain matrices $K_{p}$ and $K_{d}$, which are small. The simulation results show that the improvement of tracking accuracy is due to synchronous online incremental learning and deployment.

Figure 4b illustrates the thrust output of each propeller. It is evident that at the commencement of tracking, the RHRL controller maximally utilizes the onboard thrust capability to achieve convergence as swiftly as possible. In essence, the state remains within the prescribed boundary, aligning with expectations. It is also notable that RHRL demonstrates superior adjustment capability and undergoes more rapid adjustments.

**Figure 5:**
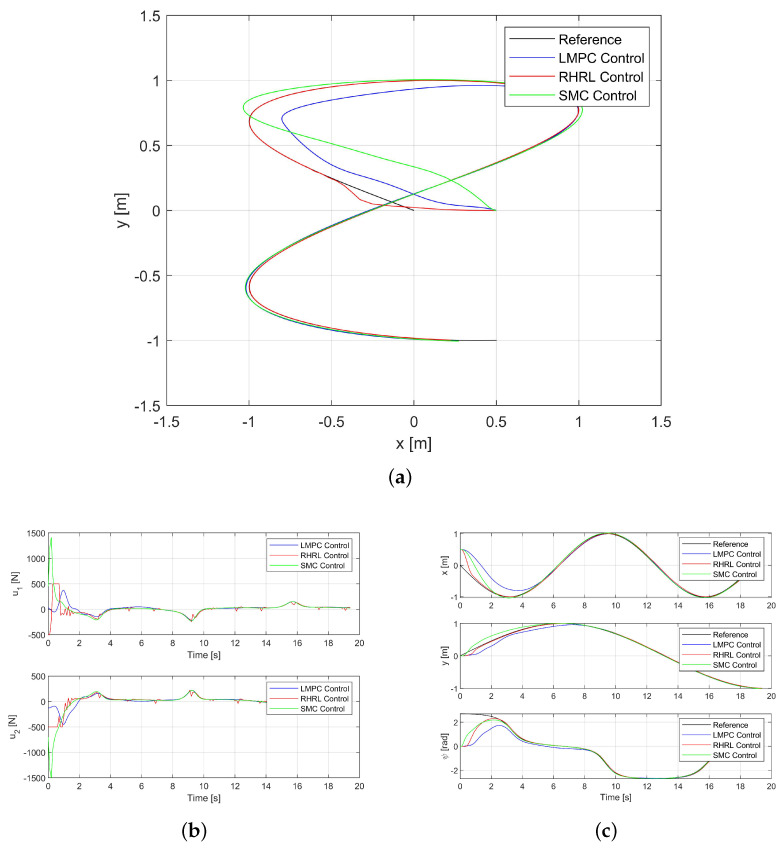
The USV trajectory tracking performance in Path II. (**a**) The USV trajectory for Path II. (**b**) The thrust outputs for Path II. (**c**) The state trajectories for Path II.

The outcomes for Path II are presented in Figure 5. Similarities arise from the observations: The USV exhibits quicker convergence to the desired trajectory through RHRL.

### 3.3. Robustness Experiment with Disturbance

The incorporation of the receding horizon implementation introduces feedback into the closed-loop system. One of the inherent advantages of the RHRL controller is its robustness toward disturbances and emergencies, making it particularly well-suited for control systems in marine and submarine environments.The RHRL’s robustness is thoroughly examined and demonstrated through simulations. The definite simulated disturbance of magnitude ${\left[100(N),100(N),0(Nm)\right]}^{T}$ was added. To provide a clearer visualization of the deviation between the three algorithms, the reference trajectory, indicated by a black line, is also included in this experiment.

**Figure 6:**
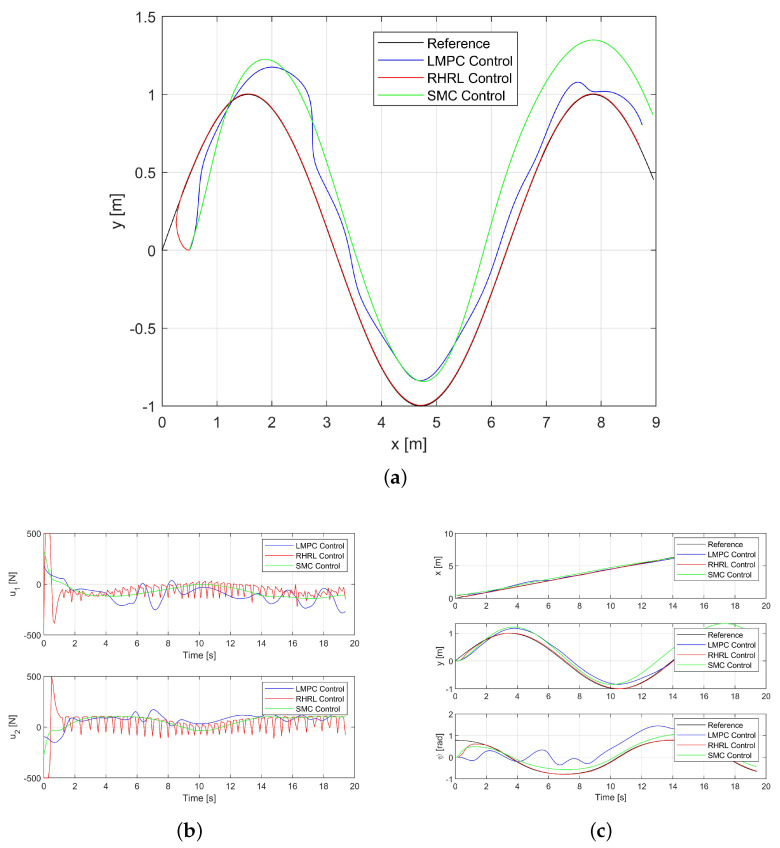
The USV trajectory tracking performance in Path I with disturbance. (**a**) The USV trajectory for Path I. (**b**) The thrust outputs for Path I. (**c**) The state trajectories for Path I.

**Figure 7:**
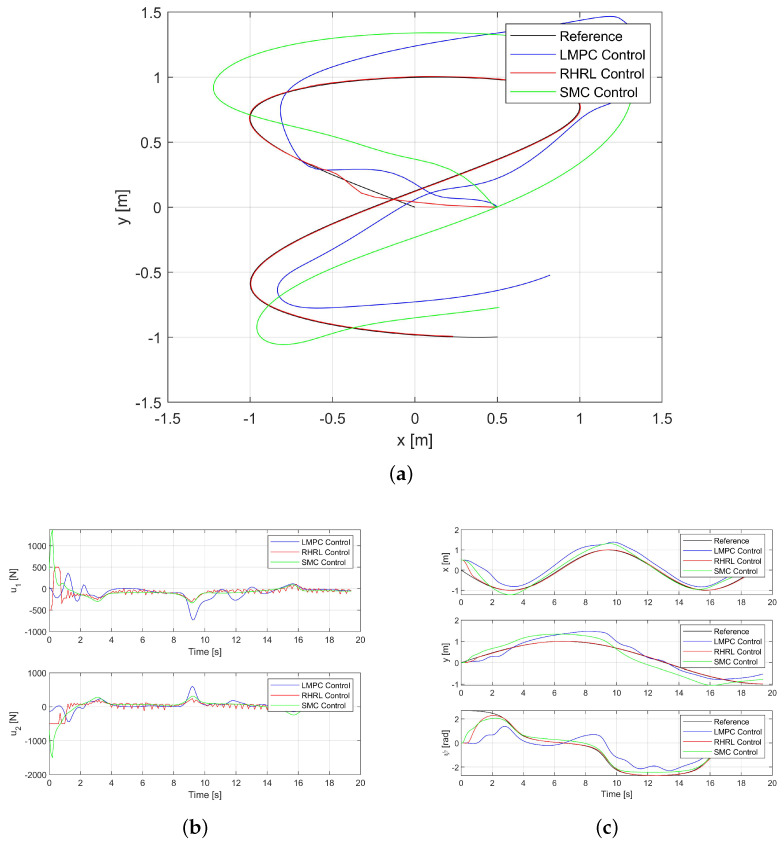
The USV trajectory tracking performance in Path II with disturbance. (**a**) The USV trajectory for Path II. (**b**) The thrust outputs for Path II. (**c**) The state trajectories for Path II.

In analyzing the outcomes shown from Figure 5 to Figure 6, it is evident that RHRL tracking control consistently guides the USV to adequately converge toward the desired trajectory. In contrast, substantial tracking errors are exhibited when conducting tracking control using LMPC, the even greater errors are associated with SMC. Figure 6b and Figure 7b illustrate that the RHRL controller consistently provides feedback for responding within a small time domain, ensuring minimal deviation.

The MSEs (mean square errors) for both paths are consolidated in Table 2 and Table 3. Generally, the MSEs are approximately 10 times smaller for RHRL compared to LMPC and SMC, especially in the case of Path II. Indeed, it is widely acknowledged that smaller MSEs correspond to reduced tracking error, thereby resulting in higher tracking accuracy; thus, it is evident that the RHRL algorithm significantly enhances tracking accuracy.

In order to more objectively demonstrate the excellent performance of the algorithm, we propose conducting quantitative analysis based on a new factor, namely thrust output. It is known that a smaller average value of thrust corresponds to lower energy consumption and enhanced cost-effectiveness. The specific data are shown in Table 4 and Table 5. As can be seen from the tables, the energy consumption of RHRL compared with LMPC is reduced by 43.85% and 41.65% for Paths I and II, respectively. The data show that RHRL is much more economical than LMPC. However, due to the algorithm characteristics, RHRL does not have a significant advantage over SMC based on this analysis.

The observed disparity stems from RHRL’s ability to learn and adapt online, utilizing online optimization to dynamically adjust control gains and effectively compensate for interference. Conversely, both LMPC and SMC lack this flexibility. Consequently, robustness is significantly enhanced by RHRL control.

## 4. Conclusions

In this paper, a trajectory control algorithm for USV based on RHRL is introduced in which reinforcement learning is seamlessly integrated with a rolling time domain optimization mechanism. Thus, infinite time self-learning optimization problems are effectively converted into a series of finite time optimization problems, which can then be solved using an executive–evaluator algorithm. The incorporation of the rolling time domain mechanism in this design approach significantly enhances the learning efficiency of the RL algorithm. Moreover, compared to LMPC and SMC, the optimization method utilizing both effector and evaluator contributes to enhanced computational efficiency. In diverging from the majority of existing finite time domain executive–evaluator learning algorithms, the proposed RHRL employs a time-independent single-network structure. This innovative approach serves to diminish the intricacy associated with network design and online computational complexity. Moreover, we analyzed the stability of the closed-loop system theoretically. Concerning scenarios involving significant errors in the learned approximation strategy, we plan to conduct in-depth analysis and substantiation in our forthcoming research. The results of simulations demonstrate that our algorithm is effective based on comparison with typical traditional algorithms in simulation scenarios. The simulation results show that RHRL control is superior to LMPC and SMC in terms of control performance and computational efficiency while also being more economical than LMPC. RHRL control also has lower sample complexity and higher learning efficiency.

## Figures and Tables

**Table 1:** Parameters for USV simulation.

Parameters	Value
*M*/kg (mass of USV)	37
*D*/m (distance from motors and center of mass)	0.7
*K* (viscosity coefficient)	0.1
*I* (moment of inertia)	0.2
$T_{e}$ (sampling period)	0.2
*i* (loop index)	1
$U_{cruise}=U_{1}=U_{2}$	2

**Table 2:** MSE for disturbances in Path I.

MSE	LMPC	RHRL	SMC	Improvement I	Improvement II
*x*/$m^{2}$	0.0518	0.0086	0.0732	83.3%	88.2%
*y*/$m^{2}$	0.0286	0.0031	0.0391	89.3%	92.1%
ψ/${\mathrm{rad}}^{2}$	0.3198	0.0358	0.4273	88.9%	91.6%

**Table 3:** MSE for disturbances in Path II.

MSE	LMPC	RHRL	SMC	Improvement I	Improvement II
*x*/$m^{2}$	0.1386	0.0158	0.1450	88.6%	89.1%
*y*/$m^{2}$	0.0968	0.0079	0.1002	91.8%	92.1%
ψ/${\mathrm{rad}}^{2}$	0.8663	0.3561	0.9984	58.8%	64.3%

**Table 4:** The average thrust output with disturbances in Path I.

TO	LMPC	RHRL	SMC	Improvement I	Improvement II
$\left|U_{1}\right|/N$	152.9	86.3	86.8	43.6%	0.57%
$\left|U_{2}\right|/N$	154.2	86.5	87.6	43.9%	1.25%

**Table 5:** The average thrust output with disturbances in Path II.

TO	LMPC	RHRL	SMC	Improvement I	Improvement II
$\left|U_{1}\right|/N$	106.7	62.1	63.2	41.8%	1.74%
$\left|U_{2}\right|/N$	109.2	63.9	64.6	41.5%	0.11%

## Data Availability

Data are contained within the article.

## Abbreviations

The following abbreviations are used in this manuscript:

USV	unmanned surface vehicle
RHRL	receding horizon reinforcement learning
LMPC	Lyapunov model predictive control
PID	proportional integral derivative
MPCC	model predictive controlling control
ADP	approximate dynamic programming
KDHP	kernel-based DHP
BF	body frame
IF	inertial frame
MSE	mean square error
